# State-dependencies of learning across brain scales

**DOI:** 10.3389/fncom.2015.00001

**Published:** 2015-02-26

**Authors:** Petra Ritter, Jan Born, Michael Brecht, Hubert R. Dinse, Uwe Heinemann, Burkhard Pleger, Dietmar Schmitz, Susanne Schreiber, Arno Villringer, Richard Kempter

**Affiliations:** ^1^Minerva Research Group BrainModes, Max Planck Institute for Human Cognitive and Brain SciencesLeipzig, Germany; ^2^Department of Neurology, Charité University Medicine BerlinBerlin, Germany; ^3^Bernstein Center for Computational Neuroscience, Humboldt-Universität zu BerlinBerlin, Germany; ^4^Berlin School of Mind and Brain & Mind and Brain Institute, Humboldt-Universität zu BerlinBerlin, Germany; ^5^Department of Medical Psychology and Behavioral Neurobiology & Center for Integrative Neuroscience (CIN), University of TübingenTübingen, Germany; ^6^Neural Plasticity Lab, Institute for Neuroinformatics, Ruhr-University BochumBochum, Germany; ^7^Department of Neurology, BG University Hospital Bergmannsheil, Ruhr-University BochumBochum, Germany; ^8^NeuroCure Cluster of ExcellenceBerlin, Germany; ^9^Clinic for Cognitive Neurology, University Hospital LeipzigLeipzig, Germany; ^10^Max Planck Institute for Human Cognitive and Brain SciencesLeipzig, Germany; ^11^Neuroscience Research Center NWFZ, Charité University Medicine BerlinBerlin, Germany; ^12^Max-Delbrück Center for Molecular Medicine, MDCBerlin, Germany; ^13^Center for Neurodegenerative Diseases (DZNE)Berlin, Germany; ^14^Department of Biology, Institute for Theoretical Biology (ITB), Humboldt-Universität zu BerlinBerlin, Germany

**Keywords:** learning, plasticity, brain scales, state-dependencies, computational modeling

## Abstract

Learning is a complex brain function operating on different time scales, from milliseconds to years, which induces enduring changes in brain dynamics. The brain also undergoes continuous “spontaneous” shifts in states, which, amongst others, are characterized by rhythmic activity of various frequencies. Besides the most obvious distinct modes of waking and sleep, wake-associated brain states comprise modulations of vigilance and attention. Recent findings show that certain brain states, particularly during sleep, are essential for learning and memory consolidation. Oscillatory activity plays a crucial role on several spatial scales, for example in plasticity at a synaptic level or in communication across brain areas. However, the underlying mechanisms and computational rules linking brain states and rhythms to learning, though relevant for our understanding of brain function and therapeutic approaches in brain disease, have not yet been elucidated. Here we review known mechanisms of how brain states mediate and modulate learning by their characteristic rhythmic signatures. To understand the critical interplay between brain states, brain rhythms, and learning processes, a wide range of experimental and theoretical work in animal models and human subjects from the single synapse to the large-scale cortical level needs to be integrated. By discussing results from experiments and theoretical approaches, we illuminate new avenues for utilizing neuronal learning mechanisms in developing tools and therapies, e.g., for stroke patients and to devise memory enhancement strategies for the elderly.

## Introduction: bridging brain scales

**Figure 1:**
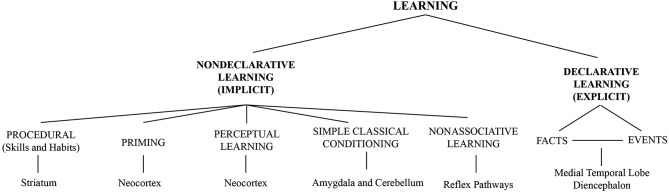
**Taxonomy of different forms of learning**. Figure taken from Sigala et al. (2014) and adapted from Squire and Zola (1996) with permission.

Learning refers to the ability of nervous systems to adapt to changing internal and external conditions, and includes perceptual processes of information uptake (encoding) as well as the storage of information. Adaptation arises from cellular and synaptic changes, i.e., synaptic plasticity (Martin et al., 2000; Malenka and Bear, 2004; Nicoll and Schmitz, 2005; Brecht and Schmitz, 2008) and through interactions of neuronal networks. Local plasticity can lead to altered representations extending over distributed cortical and subcortical networks (Roy et al., 2014b; Sigala et al., 2014). A variety of learning forms exists. They all are based on similar molecular mechanisms for altering synaptic efficiency between individual neurons. A taxonomy of learning types is displayed in Figure 1.

Prior knowledge influences neuronal dynamics and essentially contributes to the context in which learning takes place (Abraham and Bear, 1996; Abraham, 2008; Hulme et al., 2014). Ongoing cognitive processes can manifest as coherent network fluctuations in functional magnetic resonance imaging (fMRI) and as rhythmic activities in electroencephalography (EEG), which define the current state of the brain. Beyond this rhythmic activity, also non-oscillatory, arrhythmic fluctuations are observed (He et al., 2008; Nir et al., 2008). Such intrinsic dynamics shape the manner in which the brain responds and adapts to external events during waking. During sleep, certain brain states are essential for storing information for the long term (Marshall and Born, 2007; Inostroza and Born, 2013; Rasch and Born, 2013).

Neuronal rhythms regulate learning on different spatial scales, for example at a synaptic level in the hippocampus (Eschenko et al., 2008), but also in hippocampal-neocortical communication (Clemens et al., 2007). Hence specific brain states and oscillations are fundamental for learning. To exert directed influence on learning such as in teaching and rehabilitation, it is essential to know how intrinsic neuronal dynamics and learning interact. To this end, experimental tools ranging from patch-clamp recordings of awake behaving animals (Lee et al., 2006) to noninvasive measurements of neuronal population activity by simultaneous EEG-fMRI (Ritter and Villringer, 2006) and theoretical methods comprising single cell (Schreiber et al., 2004; Leibold and Kempter, 2006; Gundlfinger et al., 2007; Thurley et al., 2008) to macroscopic modeling approaches (Freyer et al., 2011a, 2012b; Ritter et al., 2013; Roy et al., 2014b; Sigala et al., 2014) need to be integrated. We will review the interplay of intrinsic states and learning in animal models and human subjects at various levels from single cells to animal and human behavior using *in-vitro* and *in-vivo* electrophysiology, imaging, neurocomputation as well as perceptual and memory paradigms.

Two major brain states are wakefulness and sleep. Whereas the wake mode of activity mainly serves the encoding and immediate processing of new information, during sleep brain oscillations are often important for the consolidation of memory, for example, by regulating long-distance communication between hippocampus and neocortex.

At a cellular level, for example the relation between hippocampal theta-rhythms with embedded gamma oscillations and sharp wave-ripples (SPW-Rs) plays an important role for the encoding of new declarative information in the wake state, and the relation between the neocortical slow oscillation (SO) and hippocampal SPW-Rs is essential for storing and consolidating this information during sleep. Therefore it is necessary to develop models of hippocampal circuitries to predict ripple-dependent alterations of cellular properties.

For perceptual learning (PL), ongoing large-scale brain dynamics associated with different states of attention interplay with learning during wakefulness. The sequence of external stimuli relative to certain brain states is relevant for perceiving these stimuli. Also the amplitude, phase, coherence and nonlinear behavior of a variety of EEG frequency bands ranging from very slow to ultra-fast rhythms influence the efficiency of passive PL. PL induced by active training requires attention mechanisms in contrast to stimulation-induced perceptual improvement. Consequently, different effects of ongoing dynamics exist. In order to reveal fundamental mechanisms underlying the diverse experimental findings at the different brain scales, computational models at various space-time scales need to be employed. Only an integrative approach will allow us to address the complex issue of the role of intrinsic neuronal activities for learning in a comprehensive and meaningful manner. By means of a number of examples we will detail the diverse aspects of this integrative work.

In the present review article, we illustrate mechanisms of explicit memory across scales reaching from sub-cellular processes to behavior. Specifically, we elucidate how theta rhythm and dendritic computations of hippocampal neurons could contribute to the encoding of space in cognitive maps. We continue on the cellular scale and review work conducted *in vitro* in rat hippocampus. This work illuminates the role of neuromodulators for the regulation of brain states that are relevant for learning of declarative memories. Next, we link cellular SPW-R activity observed invasively in rats to human behavior and report the latest insights concerning consolidation of episodic memory representations mediated by hippocampal and neocortical networks. Finally we deal with the question how intrinsic states and the temporal sequences of external events interact and lead to plasticity and learning. Here the focus is set on a form of implicit learning, namely PL. In PL, perceptual abilities improve through practice, training or pure exposure to stimulation. We address the question how does the brain know which input is relevant and what is the role of endogenous oscillations herein. In this context we highlight the clinical relevance of those issues—particularly for stroke rehabilitation.

## Dendritic computations, theta, and spatial learning in the hippocampus and the entorhinal cortex

The mammalian hippocampus plays a key role in learning and memory in general and in the formation of episodic memories in particular (Squire, 1992). Lesions of this structure in humans impair the ability to learn new facts and remember life events. Further analysis in rodents has led to the idea that hippocampal cells serve as a cognitive map representing the environment (O’Keefe and Nadel, 1978). During exploratory behaviors, hippocampal principal cells increase their firing rate and show “complex spike activity” (O’Keefe and Dostrovsky, 1971) at specific locations of an environment, that is, at their “place fields” (Fox and Ranck, 1975). However, the mechanisms underlying basic cellular phenomena, such as complex spike activity, are unclear.

Hippocampal cognitive maps are very flexible and adapt to represent new environments. The hippocampus is hence one of the most plastic structures in the brain. Accordingly, long-term potentiation (LTP) was first described in the hippocampus in the early 1970s (Bliss and Lomo, 1973). When an animal enters a new environment, place cells rapidly develop new spatial receptive fields that remain stable for weeks and months. It was suggested that place cells fire spatially tuned bursts with large, putatively calcium-mediated depolarizations that could trigger plasticity and stabilize the new map for long-term storage (Epsztein et al., 2011). An important feature of these maps is that the same place cells in the hippocampus can participate in the formation of multiple maps in different environments. Major questions regarding place cells, however, remain unanswered: How are place cells’ spatial receptive fields formed once an animal is exposed to a new environment? How can such a spatial representation in the hippocampus contribute to episodic memory?

### Development of hippocampal spatial receptive fields

Regarding the development of spatial receptive fields in the hippocampus, a working hypothesis is that the spatial adaptation of place cells results from synaptic plasticity processes such as LTP, long-term depression/LTD (Bear and Abraham, 1996), or spike-timing-dependent plasticity/STDP (Gerstner et al., 1996; Markram et al., 1997; Bi and Poo, 1998). However, the stimulation protocols that are typically used to evoke synaptic plasticity *in vitro* cannot explain the *in vivo* plasticity of place cells because: (1) LTP protocols require a high stimulation frequency that is far beyond firing frequencies of place cells *in vivo*; (2) STDP protocols, which are closer to *in vivo* activity, and LTD protocols require a large number of repetitions of stimuli, and the time required to deliver a sufficient number of repetitions is too long; therefore *in vivo*-like plasticity stimuli, e.g., (Gundlfinger et al., 2010) pose a problem with regard to the speed of change of place cells observed when an animal enters a new environment. Thus, present plasticity models do not suffice to explain place-cell formation.

An alternative hypothesis trying to unravel the mechanisms of formation and maintenance of hippocampal place cells involves dendrites. Neurons in the central nervous system typically possess elaborated dendritic trees, which receive and integrate input. Understanding dendritic integration is central to our understanding of how the brain processes the vast synaptic input. In recent years, through the development of new visually guided voltage recordings and imaging techniques *in vitro*, it became evident that dendrites of neocortical and hippocampal pyramidal neurons contain a large variety of voltage-gated channels that can considerably influence the integrative properties of a neuron. For reviews see (Johnston et al., 1996; Yuste and Tank, 1996; Magee et al., 1998; Häusser et al., 2000; Reyes, 2001; London and Häusser, 2005). Active dendritic conductances can generate local dendritic spikes, which may increase the computational capabilities of neurons by mediating nonlinear integration (Hausser and Mel, 2003; Poirazi et al., 2003; Polsky et al., 2004).

The role of dendritic morphology and excitability in the intact hippocampus was unclear until a few years ago because the available recording methods did neither identify cellular morphology nor did they allow the recording of dendritic activity in awake, behaving animals. However, recently a new method emerged that may in the future facilitate dendritic recordings from identified cells in the hippocampus of freely moving rodents (Lee et al., 2006). This recording method for the first time provided data that can be interpreted in a computational framework of hippocampal representations.

Recording the somatic membrane voltage of hippocampal pyramidal cells in freely behaving rats, (Lee et al., 2012) showed how place fields can suddenly emerge. A small and constant depolarization of the membrane potential led to the instantaneous emergence of a place field. This effect was reversible, which pointed to gating of dendritic inputs (Major et al., 2013). To further test this hypothesis, (Sheffield and Dombeck, 2014) used two-photon imaging and recorded dendritic and somatic calcium transients in place cells of awake behaving mice. Local dendritic spikes, which can amplify synaptic input, predicted the spatial precision and persistence or disappearance of place fields. These findings confirm that the nonlinear dynamics in dendritic trees is essential for the formation of a spatial representation in the hippocampus. It is therefore also feasible that dendritic spikes provide the post-synaptic signal for Hebbian plasticity, which is in contrast to basic models of place cells that rely on linear neuronal integration.

The plasticity of spatial receptive fields of hippocampal neurons *in vivo* may thus depend on local dendritic spikes, and synaptic potentials could undergo quasi-instantaneous potentiation. It is interesting to note that *in vitro* studies indicate an almost instantaneous induction of LTP that is mediated by dendritic spikes (Huerta and Lisman, 1995; Holthoff et al., 2004). Active dendritic conductances may therefore control long-term synaptic plasticity (Golding et al., 2002; Gordon et al., 2006; Letzkus et al., 2006). We also note that plasticity *in vivo* depends on brain states, too. As pointed out in Section Role of Neuromodulators on Theta, Gamma Oscillations, Sharp Wave Ripple Activity and LTP in Hippocampus, neuromodulators such as acetylcholine (Ach) are essential in controlling both the oscillatory state and the plasticity in the hippocampal formation (Hasselmo, 2006).

### Contribution of spatial representations in the hippocampus to episodic memory

How can hippocampal place fields contribute to episodic-like memory? As a simple example of an episode we consider a spatial trajectory, which is an ordered sequence of places that activates corresponding place cells in the hippocampus. The storage or “encoding” of such a sequence of place-cell activities might be achieved by strengthening of synapses that connect neighboring, overlapping place fields. However, there are major problems with such an approach: The time scale of the sequence of places (seconds) does not match the time scale of plasticity models such as STDP (tens of milliseconds) and it is unclear how the temporal order of places can be stored. To explain a possible solution to these problems, we first focus on the oscillatory state of the hippocampus during the awake state and then explain a phenomenon called “phase precession”.

Complex behaviors such as spatial exploration in rodents, working memory in primates, and navigation and working memory in humans are consistently associated with theta rhythms, i.e., 4–12 Hz oscillations (Buzsáki and Draguhn, 2004). This oscillation is coherent in the whole hippocampal formation and the entorhinal cortex (EC). Furthermore, the action potentials of most hippocampal cells, including “place cells”, are coordinated to the ongoing theta rhythm, that is, cells discharge at specific phases of the theta rhythm. This locking of pre- and postsynaptic spikes to a particular phase of the theta cycle can boost plasticity because spikes occur with higher probability in close (about 10 ms) temporal proximity. However, the temporal order of place fields cannot be preserved with simple phase locking.

Another basic phenomenon observed in the hippocampal formation and the EC is “phase precession”. As outlined above, spikes that are generated within a firing field can be assigned a phase with respect to the theta oscillation in the local field potential, the latter of which is prominent when an animal moves. Moreover, during the traversal of a firing field of a principal cell, the phase of spiking has been shown to precess, so that spikes occur at earlier and earlier phases of the local-field theta. In other words, within a firing field, the phase (relative to the theta oscillation) of spikes decreases as a function of position (O’Keefe and Recce, 1993; Hafting et al., 2008; Schmidt et al., 2009). Interestingly, to match the time scales of plasticity, phase precession can be used to temporally compress behavioral sequences while preserving the temporal order of places visited (Skaggs et al., 1996). In general, phase precession increases the spatial information (Jensen and Lisman, 2000; Reifenstein et al., 2012, 2014).

To test the above-mentioned hypothesis how an episodic memory trace may be encoded, one could interfere with phase precession. (Robbe and Buzsáki, 2009) described a profound effect of cannabinoids on memory and navigation, which correlated with reduced phase precession. Further behavioral correlates of altered phase precession are not available, which is mainly due to the observation that it is difficult to change phase precession without affecting place-field activity (Zugaro et al., 2005; Royer et al., 2012). Another reason may be that the mechanisms underlying phase precession are unknown, although a variety of computational models have been proposed to explain its generation. Potential mechanisms include: (1) the interaction of two oscillators with slightly different frequencies, e.g., (O’Keefe and Recce, 1993; Kamondi et al., 1998; Lengyel et al., 2003); (2) the asymmetry of place fields (Mehta et al., 2000, 2002); (3) the adaptation of the spiking activity of pyramidal cells in response to sustained excitatory input (Kamondi et al., 1998; Magee, 2001; Harris et al., 2002; Mehta et al., 2002); (4) a short-term memory buffer (Hasselmo et al., 2002; Koene et al., 2003); (5) short-term facilitation (Thurley et al., 2008); (6) interaction of neurons in recurrent networks (Jensen and Lisman, 1996; Tsodyks et al., 1996; Wallenstein and Hasselmo, 1997; Baker and Olds, 2007; Navratilova et al., 2012); (7) coupling of excitatory and inhibitory neurons (Bose et al., 2000; Castro and Aguiar, 2012; Cutsuridis and Hasselmo, 2012); (8) integration of properly tuned inputs (Geisler et al., 2010; Chance, 2012; Thurley et al., 2013); and (9) inheritance (Jaramillo et al., 2014). In these models, the seminal importance of specific properties of synapses, dendrites, neurons, and the network has been emphasized to varying degrees. Even the brain region where phase precession is generated differs from model to model and ranges from the EC to the Cornu Ammonis areas 3 (CA3) and area 1 (CA1) subregions of the hippocampus. Finally, some models of phase precession stress its role for encoding, e.g., (Thurley et al., 2008) whereas others propose phase precession as a result of the retrieval of memories, e.g., (Tsodyks et al., 1996).

We are only beginning to understand mechanisms underlying basic cellular phenomena *in vivo*, and a considerable amount of further research is required to unravel dendritic computations and their role in learning and memory. In this section we have reviewed mechanisms related to storage, representation, and retrieval of declarative memories in the hippocampus during the awake state. Further progress will depend on recordings from dendrites, an evaluation of the specific rules of plasticity of synapses at various dendritic locations, and a test of computational mechanisms underlying temporal coding. This new data might allow us understanding the mechanisms by which internal spatial cognitive maps are adapted to new environments. It may also enable us to link plasticity mechanisms at the level of dendrites to changes that take place at the network- and ultimately the cognitive level. In subsequent sections we connect this line of research on the awake state to other states of the brain such as sleep, where consolidation of declarative memories is important (Epsztein et al., 2011).

## Role of neuromodulators on theta, gamma oscillations, sharp wave ripple activity and LTP in hippocampus

### Two-stage theory of formation of explicit memory

The two-stage theory of formation of explicit memory says that during explorative behavior transient memory traces are formed in the hippocampus. However, as long-term storage of memory is not hippocampus dependent, any information has to be transferred from the hippocampus into the cortical mantle in a subsequent process termed memory consolidation. This process is believed to depend on the generation of SPW-R complexes during which previously stored information is read out in a temporally compressed form characterized by high-frequency network oscillations of 120–200 Hz (Buzsáki et al., 1992; Axmacher et al., 2006, 2008; Born, 2010). SPW-Rs might support the induction of LTP in remote areas and thus may serve for memory consolidation.

### Hippocampal sharp wave ripple activity

Exploratory behavior in rodents—i.e., perception/encoding of information—is characterized by pronounced theta in the hippocampal formation with embedded gamma oscillations; consummatory behavior—i.e., memory storage/consolidation—is related to the appearance of SPW-Rs (Chrobak and Buzsáki, 1996; Axmacher et al., 2006). Transition between these two states of activity can be rather abrupt. SPW-R activity can be blocked when a novel situation requires reorientation such as sudden changes in the environment or in body state requiring changes of attention and behavior.

### Systemic neuromodulation

Systemic neuromodulators regulate the different modes of network activity. They exert influence via neurons that send their fibers to large parts of the cortical mantle and, when activated, release substances such as ACh, norepinephrine (NE), dopamine or serotonin into many parts of the brain. Systemic neuromodulation implies in this context not only that agents affect locally a set of neurons. Rather they affect the way in which neuronal assemblies are temporally formed and how interactions between sets of neurons are enabled. This is achieved by a concerted modulation of excitability and release properties of GABAergic and glutamatergic synapses. Global modulation of network activity may be locally controlled by substances that are released by individual sets of neurons. These include adenosine, adenosine triphosphate (ATP; Schulz et al., 2012) and probably also some peptides (Decker et al., 2009) and involve spillover of glutamate and GABA from the synaptic cleft thereby mediating volume transmission through high affinity ionotropic and metabotropic receptors (Hollnagel et al., 2014).

Theta and gamma are typical for exploratory behavior with gamma oscillations being a factor in determining windows for STDP (Yoshioka, 2002). Effects of global neuromodulators on oscillatory network activity have been studied. ACh in the presence of physostigmine can induce theta and gamma in *in vitro* hippocampal slices (Wójtowicz et al., 2009; Fano et al., 2011). In relation to regulation of wakefulness and sleep, ACh can cause release of histamine and thereby augment gamma (Fano et al., 2011). Gamma is immediately suppressed by monoamines such as serotonin, dopamine and NE. In case of dopamine, the effect seems to be dependent on D1 receptor activation and to involve up-regulation of cyclic adenosine monophosphate (cAMP; Weiss et al., 2003; Wójtowicz et al., 2009). This effect was restricted to gamma oscillations in the low-frequency range (≈40 Hz) and did not apply to gamma oscillations that occur shortly after a stimulus. The latter are augmented by NE, dopamine and serotonin. This suggests that neuromodulators, which affect many regions of the brain simultaneously, do alter network interactions and thereby change possibilities for spatial interaction in the brain. In addition they may prepare the brain for storage of new information as indicated by facilitated induction of LTP in synapses that are specifically activated in a given mental process and thereby tagged for formation of neuronal ensembles when required.

Therefore potential effects of neuromodulators on stimulus-induced SPW-Rs moved in the focus. Induced SPW-Rs mimic spontaneous SPW-Rs observed in rodents during immobility and slow-wave sleep/SWS (Maier et al., 2003; Behrens et al., 2005). It was suggested that during such events ensembles of neurons are activated, which replay previously stored events on a compressed time scale and thereby may be involved in transfer of information from the hippocampus to other regions of the brain and hence in memory consolidation. Protocols inducing LTP in the hippocampus can also induce SPW-R when protocols for induction of late or long-lasting LTP are employed. Therefore the effects of monoamines on the induction and maintenance of SPW-R have been studied. A candidate substance was NE. NE is released when the locus coeruleus gets activated. It releases NE over wide areas of the cortex including the hippocampal formation. The substance has been early recognized to be involved in controlling network activity for example in epileptic animals and patients (Stanton et al., 1992). NE was further reported to be involved in facilitation of LTP induction—an effect that is mediated by ß receptors of NE involving also facilitated activation of N-methyl-D-aspartate (NMDA) receptors. Interestingly, ßreceptor agonists of NE facilitated induction not only of LTP but also of SPW-R complexes (Ul Haq et al., 2012). When testing for effects of NE alone, LTP was induced but SPW-R did not appear. SPW-R became apparent only after the application of NE. This indicates that during induction of LTP certain synapses increase coupling efficacy but are not yet ready to integrate their activity into a neuronal ensemble. NE blocked stimulation induced SPW-R through activation of alpha-1 receptors for NE (Ul Haq et al., 2012). The underlying mechanism seems to be related to an alteration in presynaptic release probability mediated by effects on transmitter release. In case of NE it was shown that alpha agonists of NE and NE itself would alter paired pulse indices and presynaptic Ca^2+^ uptake indicating alterations in presynaptic release probability. If this scenario applies generally, neuromodulators can set neuronal interaction modes in two ways: on the one hand by facilitating or depressing induction of LTP and on the other hand by regulating on a short time scale the probability of transmitter release, which means that in absence of such neuromodulator the likelihood increases that neurons organize themselves into an ensemble. Preliminary evidence suggests that serotonin may have a similar role. In addition also local neuromodulation may determine whether such interactions occur as recently shown for cannabinoids and adenosine (Maier et al., 2012; Schulz et al., 2012).

Effects of Ach on SPW-R were examined, too. As was the case with NE, ACh immediately interrupts stimulus induced SPW-Rs but other than NE replaced this activity by gamma activity. Again this interruption may depend on regulation of transmitter release from presynaptic endings and/or depolarization of specific sets of interneurons. ACh was previously shown to facilitate induction of LTP and it was therefore of interest to test whether LTP producing protocols would permit switching from gamma activity to SPW-Rs which by elevating ACh could then be replaced by gamma activity again. Recent studies indicate that gamma oscillations result in strong consumption of oxygen (Huchzermeyer et al., 2008) and correlate best with areas in which blood flow is increased. This would suggest that wide spread synchronization of selected parts of the brain is essential for cognition and learning. Whether there is a neocortical equivalent of SPW-Rs is still an open question but it was suggested that sleep spindles might be such an equivalent (Born et al., 2006), see Section Interactions Between Intrinsic Activity and External Triggers in Perceptual Learning.

## Slow oscillations to coordinate system consolidation of hippocampal memories in sleep

### Wakefulness and sleep in episodic memory

Whereas new information is primarily encoded during wakefulness, sleep is the brain state serving primarily the consolidation of newly encoded memories (Stickgold, 2005; Rasch and Born, 2013; Stickgold and Walker, 2013). This does not mean that memories cannot stabilize and consolidate in the wake state. Synaptic consolidation is probably achieved during wakefulness in the same way as during sleep (Dudai, 2004). Rather, sleep appears to favor a unique type of system consolidation that involves the reactivation of the newly encoded memory representations and their redistribution to networks serving as long-term store, and differs from reactivation-induced processes of system consolidation that are observed during wakefulness (Diekelmann et al., 2011). Also, consolidation during sleep does not appear to equally affect all types of memory but, in particular, memories involving the hippocampal system at encoding, i.e., memories that bear episodic features (Albouy et al., 2013a,b; Inostroza and Born, 2013), although this is a matter of ongoing debate, e.g., (Stickgold and Walker, 2013). Consolidation of such episodic memories benefits in particular from SWS that is thought to promote the redistribution of respective hippocampal representations to extrahippocampal, preferentially neocortical and striatal circuitry (McClelland et al., 1995; Ackermann and Rasch, 2014). This system consolidation process implicates a reorganization of the memory representation such that relevant and invariant features become extracted from the episodic memory to be integrated within pre-existing schemata and semantic knowledge (Lewis and Durrant, 2011; Kroes and Fernández, 2012). System consolidation during SWS relies on a concerted action of brain EEG rhythms, the exact features of which are still being explored. The consolidation of memory during sleep is state-dependent as it requires the presence of <1 Hz EEG SOs that are hallmarks of SWS and do orchestrate the consolidation process.

### Role of slow oscillations during sleep

In humans, SO’s occur in non-rapid eye movement (non-REM) sleep-stage 2 and SWS with a peak frequency of 0.75 Hz (Achermann and Borbély, 1997). They are generated in neocortical networks, in part as a function of the prior use of these networks for encoding of information; i.e., the more information is encoded during the wake phase the higher the amplitude is of the SOs generated in respective neocortical circuitry during subsequent SWS (Huber et al., 2004; Mölle et al., 2004; Tononi and Cirelli, 2014). The SO temporally groups neocortical activity into down states of neuronal silence where neurons are globally hyperpolarized, and up states of strongly enhanced excitation resulting from global membrane depolarization (Steriade, 2003). Via efferent projections the depolarizing up state of SOs drive the generation of (12–15 Hz) spindles which originate from thalamic nuclei and reach, via thalamocortical projections widespread areas of the neocortex. Like SOs, spindle activity has been implicated in the consolidation of memories during sleep (Gais and Born, 2004; Fogel and Smith, 2011). Spindle activity co-occurring with depolarizing SO up states has been proposed to induce, via activation primarily of T-type Ca^2+^ channels, a massive influx of Ca^2+^ into neocortical pyramidal cells that, via activation of Ca^2+^ sensitive kinases, can mediate long-term plastic synaptic changes underlying the formation of persistent neocortical memory representations (Rosanova and Ulrich, 2005; Destexhe et al., 2007; Marshall and Born, 2007; Ayoub et al., 2013).

### Consolidation of episodic memory: a dialog between hippocampus and neocortex orchestrated by SOs

A growing body of findings supports a concept that the consolidation of episodic memory representations (spanning hippocampal and neocortical networks) evolves from a dialog between neocortex and hippocampus such that the neocortical SOs drive in parallel the generation of thalamocortical spindles as well as hippocampal SPW-Rs, with the latter accompanying the reactivation of new memory information in hippocampal networks (Buzsáki, 1998; Diekelmann and Born, 2010). This enables the feedback from these structures, i.e., the thalamo-cortical spindles as well as the hippocampo-to-neocortical memory reactivations, enwrapped in ripples, arrives at neocortical circuitries at the same time. It is this synchrony of the memory-related inputs fed back from the thalamus and hippocampus that is considered critical to the persistent redistribution of hippocampal memories to respective neocortical networks, such that at a later stage the memory is represented preferentially in extra-hippocampal, mainly neocortical networks.

Central to this concept is the assumption that memory consolidation during sleep relies on the repeated reactivation of neuronal networks previously engaged in encoding the information. Indeed, temporal patterns of neuronal reactivation have been consistently observed during SWS after hippocampus-dependent tasks in the rat hippocampus (Wilson and McNaughton, 1994; Ribeiro et al., 2004; Euston et al., 2007; Ji and Wilson, 2007). Neuronal reactivations in the hippocampus slightly precede those in other brain regions including striatum and neocortex (Ji and Wilson, 2007; Lansink et al., 2009). In the hippocampus, reactivations occur during ripple events typically forming SPW-R complexes that originate from strong depolarization of CA3 collaterals (Buzsáki, 1996; Buzsaki, 2006). Disruption of ripples by electrical stimulation impairs consolidation of hippocampal memories (Girardeau et al., 2009). Studies in healthy humans provided direct evidence for a causative role of reactivations of hippocampus-dependent memories during SWS for memory consolidation (Rasch et al., 2007; Rudoy et al., 2009). In the former study, participants who learnt a spatial memory task in the presence of an odor showed an enhanced memory for the spatial task stimuli at a recall test after sleep, when this odor was re-exposed to the subject during SWS after learning. The reactivation of the spatial memories induced by this odor-cuing procedure was not effective with odor re-exposure taking place during REM sleep or wakefulness. Likewise, the odor was ineffective when at learning it was associated, not with a hippocampus-dependent task, but with a procedural finger tapping task. Functional MRI studies confirmed that odor re-exposure during SWS after spatial learning selectively re-activates (left) hippocampal regions. A recent study confirmed the state dependency of the consolidating effect of reactivations (Diekelmann et al., 2011). In this study, odor-induced reactivations during SWS produced an immediate stabilization of memories whereas reactivations induced in the same way during wakefulness produced a transient weakening of the memories.

Reactivation of memories during SWS co-occurring in hippocampus and neocortex has been proposed as a central mechanism promoting the redistribution of the newly acquired memories to preferential representation in neocortical networks (Ji and Wilson, 2007; Rasch and Born, 2008; Lewis and Durrant, 2011). The neocortical SO has been proposed in this context to provide a temporal frame for this hippocampo-to-neocortical information transfer. Indeed, the depolarizing up-state of the SO, in parallel to its driving influence on neocortical excitability and on thalamic spindle activity, also impacts via EC neuronal activity in the hippocampus, which does not develop slowly oscillating up and down-states on its own (Sirota and Buzsáki, 2005; Isomura et al., 2006; Mölle et al., 2006; Clemens et al., 2007). SPW-R events and CA1 interneuron activity are suppressed during SO down-states and show a rebound during development of up-states, with cortical up and down-states leading the temporal dynamics in hippocampal activity by 30–50 ms. Altogether, these observations suggest a scenario where the neocortical SO, by repeatedly resetting networks during the hyperpolarizing down-phase, sets a temporal frame that synchronizes the generation of thalamo-cortical spindles with SPW-R events occurring in hippocampal circuitry during memory reactivation. SPW-Rs that in this way become synchronized to the occurrence of a spindle may form, together with the spindle, “spindle-ripple events” where ripples and reactivated memory information nest in the excitable troughs of the spindle oscillation. Such spindle-ripple events might be a mechanism that promotes the transfer of hippocampal memory information to the neocortex and, thus, eventually the redistribution of hippocampal memories to longer-term neocortical stores (Siapas and Wilson, 1998; Marshall and Born, 2007; Mölle and Born, 2011).

Two key issues arise from this concept: (1) Do neocortical SOs indeed exert a causal top-down control such that they drive memory-reactivations and associated hippocampal ripples as well as thalamo-cortical spindles? (2) Do SOs induced ripple-spindle events conversely represent a bottom-up mechanism that facilitates the hippocampo-to-neocortical transfer of memory information to neocortical sites? So far, these questions cannot be definitely answered. Nonetheless, a causative role of the SO for hippocampus-dependent memory consolidation has been suggested by findings after experimental induction of SOs through transcranial electrical stimulation (Marshall et al., 2004, 2006). Humans stimulated during periods of SWS-rich early nocturnal retention sleep with slowly oscillating (at a frequency of 0.75 Hz) electrical potential fields showed an enhanced retention of hippocampus-dependent memories for word-pairs the subjects had learned before the sleep period, whereas memories not essentially depending on hippocampal function remained unaffected (Marshall et al., 2006). The oscillating stimulation was applied via electrodes attached bilaterally over the prefrontal cortex and to the mastoids, with maximal current densities such that the estimated potential fields produced in underlying neocortex were about the same magnitude as those naturally occurring during endogenous SOs. The stimulation was applied during early SWS-rich sleep, when the brain is prepared to generate SOs itself. The improving effect of oscillating transcranial stimulation on the consolidation of hippocampus-dependent memory was associated with distinctly enhanced endogenous SO and frontal spindle activity, indicating that stimulation induced resonance in thalamo-cortical networks responding with increased coalescent SO/spindle activity. The effect was specific for the stimulation frequency, inasmuch stimulation at theta frequency (5 Hz) did not enhance but globally suppressed endogenous SO activity and also suppressed frontal slow spindle activity (Marshall et al., 2011). Fittingly, this suppression was associated with a diminished retention of word-pairs. Finally, the effect of electrical stimulation oscillating at the 0.75 Hz SO frequency proved to depend also on the brain state (Kirov et al., 2009). Applying the stimulation protocol in waking participants, rather than during early nocturnal SWS-rich sleep, induced a profound increase in EEG theta activity rather than in SO activity. The increase in theta activity was accompanied by a significant improvement in the encoding of word memories, while consolidation processes remained unaffected. In combination, these data suggest that the cortical networks that are used during wakefulness for the up-take of hippocampus-dependent episodic information, thereby producing theta activity, are functionally linked to the networks that are used during succeeding periods of SWS to consolidate this memory and thereby produce SOs. A top-down control of the SO on thalamocortical spindles was also demonstrated in experiments using closed-loop auditory stimulation to induce SOs (Ngo et al., 2013). In these experiments, low intensity clicks distinctly enhanced SO amplitudes and prolonged trains of SOs during SWS, but only when these clicks were presented in-phase with an online detected SO. The effect was accompanied by enhanced fast spindle activity occurring during the SO upstate as well as by an improved retention of declarative word-pair memories.

### Evidence for the ripple-spindle theory

Evidence for ripple-spindle events causally contributing to the transfer of memory information from hippocampal to neocortical sites and to long-term storage of this information remains indirect. Learning of hippocampus-dependent tasks like word-pair associates in humans and odor-reward association in rats, prior to sleep, increases thalamocortical spindle activity and hippocampal ripple activity. These increases have been shown to be associated with an improved retention of respective memories (Gais et al., 2002; Eschenko et al., 2006, 2008). Moreover, SOs have not only been consistently shown to temporally group spindle activity and, in parallel, also hippocampal ripple activity in rodents and humans (Mölle et al., 2006; Clemens et al., 2007), but these studies also revealed a strikingly sharp suppression of spindle and ripple activity during the hyperpolarizing down-state of the SO suggesting that the down-state acts to induce a timed resetting of networks. Importantly, studies in rats and human epileptic patients revealed that spindles co-occurring with ripples during the transition into the SO up-state tend to form spindle-ripple events where ripples become nested into the succeeding spindle troughs (Siapas and Wilson, 1998; Clemens et al., 2011). The human studies moreover indicated that such events are specifically formed with the classical fast (12–15 Hz) spindles which predominate over centro-parietal cortical areas and, in these studies, were also observed in intracranial recordings from parahippocampal regions. Slow spindles (10–12 Hz), which concentrate over the frontal cortex, did not show a close temporal link to ripples. In fact, unlike fast spindle activity which is synchronized to the SO upstate, slow frontal spindles preferentially occur 400–500 ms later in the SO cycle, i.e., during the transition into the down-state (Molle et al., 2011), and appear to be associated with inhibited hippocampo-to-neocortical information flow (Peyrache et al., 2011). Magnetencephalographic (MEG) recordings in healthy humans revealed that, in addition to hippocampal ripples, fast spindles during sleep also phase-lock neocortical >30 Hz gamma-band activity (Ayoub et al., 2012). Gamma band activity is an indicator of coherent information processing going on in local neocortical circuitry, and also of synaptic potentiation in these networks. Collectively, these data well agrees with the notion that during the depolarizing up-state of the SO, ripples co-occur with fast spindles and form spindle-ripple events whereby ripples together with the reactivated hippocampal memory information enwrapped in the ripple, become nested into the excitatory phase of the spindle cycle. The reactivated memory information, arriving in this way at neocortical sites, may stimulate local synchronized gamma activity as a sign of integrative processing of this information that eventually sets the stage for more enduring plastic changes underlying the storage of this information in neocortical networks. Yet, the causality of the suggested spindle-ripple mechanism remains to be demonstrated.

## Interactions between intrinsic activity and external triggers in perceptual learning

While its effectiveness is well established, a central question remains: How does a neural system know, which information is behaviorally relevant and which is not? What are the mechanisms that select what is learned? The events from which we learn are organized within a complex temporal structure. Accordingly, one mechanism that could solve the problem of selecting appropriate items in a stream of inputs with a complex temporal structure is to use temporal interferences between external timing and endogenous rhythms that are characteristic for different brain states (Roy et al., 2014a; Sigala et al., 2014). We here address the role of the interaction between the timing of stimulus presentation and ongoing brain states for learning.

### Role of training for skill learning

Achieving high-level skills requires training, which is thought to optimally engage neuronal plasticity mechanisms. For example, intense practicing for tens of thousands of hours is required to develop the musical skills typically observed in professional musicians or to exhibit expert performance in sports. The use of non-invasive imaging techniques has enabled investigating the impact of intense practice and training at functional and neuroanatomical levels (Draganski et al., 2004; Yotsumoto et al., 2008; Kahnt et al., 2011; May, 2011; Shibata et al., 2011; Baldassarre et al., 2012; Jehee et al., 2012; Shibata et al., 2012).

PL—a form of implicit or non-declarative learning (Figure 1)—is not achieved by a unitary process (Sagi and Tanne, 1994; Goldstone, 1998; Fahle, 2002a). There are peripheral, specific adaptations and more general, strategic ones. There are mechanisms driven by feedback and reward (Pleger et al., 2008, 2009) and those that operate on the statistical structure of the stimuli (Preuschhof et al., 2010; Karim et al., 2012; Thiel et al., 2014). It has been shown that tactile PL is associated with selective changes in primary somatosensory cortex (S1; Recanzone et al., 1992).

### Temporal interactions of internal and external events

Cortical changes induced by PL are only partially understood. There are distinct temporal phases in the time course of PL where cortical representations first expand and then come back to normal (Yotsumoto et al., 2008). Attention plays a key role in the regulation of PL (Ahissar and Hochstein, 1993; Sarter et al., 2001), but PL can occur also as a result of mere exposure (Godde et al., 2000, 2002; Pleger et al., 2001, 2003; Watanabe et al., 2001; Dinse et al., 2003; Seitz and Watanabe, 2003; Tegenthoff et al., 2005). It has been suggested that for PL to occur, long-term sensitivity enhancements to task-relevant or irrelevant stimuli develop as a result of timely interactions between intrinsic/endogenous/top-down signals, i.e., internally triggered neuronal activity and signals produced by the external stimulus (Seitz and Watanabe, 2005). The proposed mechanism uses multiple attentional and reinforcement systems that rely on different underlying neuromodulators (see Section also Slow Oscillations to Coordinate System Consolidation of Hippocampal Memories in Sleep). In addition to attention and reinforcement, saliency and optimized sensory inputs are assumed to drive internal learning and reinforcement signals (Seitz and Dinse, 2007). Recent studies demonstrated that rhythmic stimulus sequences are required to enable PL (Kuai et al., 2005; Zhang et al., 2008). Learning was facilitated when stimuli were presented in fixed temporal sequences rather than in random order. The authors concluded that rhythmic stimulus presentation, especially with evenly spaced trials, would allow the observer to accurately switch attention to the outputs of the most appropriate set of neurons when the observer learns multi-level stimuli. It has been suggested that attention in auditory perception is an inherently oscillatory process with adjustable periodic pulses, and that a system’s responses reach maximal accuracy when attention pulses synchronize with the rhythm of the external stimuli (Large and Jones, 1999).

It is generally agreed that processes modifying synaptic efficacy are the neural substrates of learning. Studies on synaptic plasticity use temporally specific stimulation protocols to induce long-lasting changes in synaptic transmission, but the implications of this requirement for temporally specific protocols in everyday learning remain unclear (Andersen, 2011; Beste et al., 2011; Dinse et al., 2011; Clapp et al., 2012; Cooke and Bear, 2012). To induce training and practice-based learning sensory inputs are modified in their frequency, temporal pattern, the number of stimuli and their duration, form, size and intensity. But it is difficult to exactly quantify the changes in different input parameters that occur during training. Therefore, linking the principles of synaptic learning that induce plasticity at the cellular level to the principles at the systems level is far from being straightforward (Roy et al., 2014a; Sigala et al., 2014).

### Exposure based learning

This limitation can be overcome by what we shall refer to as “training-independent sensory learning”. Numerous investigations have demonstrated that human perception and behavior can change without training, simply via exposure to sensory stimulation protocols for a few minutes to a few hours (Pleger et al., 2001, 2003; Dinse et al., 2003, 2005, 2011; Seitz and Dinse, 2007; Ragert et al., 2008; Gutnisky et al., 2009; Beste et al., 2011; Clapp et al., 2012; Cooke and Bear, 2012). All these investigations have taken the approach of directly influencing human perception and behavior by using stimulation protocols known to induce plastic changes at the cellular level. The idea is to translate protocols that induce plasticity at a cellular level into sensory stimulation protocols.

### Long-term potentiation and long-term depression

From cellular studies, LTP and LTD of synaptic transmission are the leading candidates for being the relevant activity-dependent changes in synaptic connection strength (Bliss and Lomo, 1973; Stanton and Sejnowski, 1989; Bliss and Collingridge, 1993; Nicoll and Malenka, 1995; Abraham and Williams, 2003). Typically, high-frequency stimulation (10 Hz or higher) is used to induce LTP in brain slices, whereas LTD can be reliably evoked by low-frequency stimulation of around 1 Hz (Bliss and Collingridge, 1993). However, the lack of adequate input stimuli for the induction of LTP and LTD in humans has hindered direct evaluation of the impact of such protocols on human behavior. Training-independent sensory learning is based on specific stimulation protocols derived from our knowledge about celuular mechanisms of plasticity (Dinse et al., 2011). Training-independent sensory learning offers complete control of the timing and spatiotemporal allocation of the stimulation and thus is a means to systematically determine the appropriate timing for the induction of perceptual and cortical changes in humans.

### Challenge of terminology

One problem when trying to build bridges between different disciplines and fields is a different use of terminology. Yet as we demonstrate using an example form the field of PL, such inconsistent use of language also occurs within narrow fields. Hence one important task is to strive for standardized terminology or at least be aware of the different implications it may have for different scientists. In our example different laboratories are using different terms to refer processes that are essentially comparable, such as: “peripheral nerve stimulation” (Hummel and Cohen, 2006), “somatosensory stimulation” (Wu et al., 2006), “exposure-based learning” (Gutnisky et al., 2009; Beste et al., 2011), “co-activation” (Godde et al., 2000; Pleger et al., 2001; Dinse et al., 2003; Freyer et al., 2012a, 2013), “unattended-based learning” (Dinse et al., 2005), “repetitive sensory stimulation” (Kalisch et al., 2008; Dinse et al., 2011; Roy et al., 2014a; Sigala et al., 2014) and “high-frequency stimulation” (Ragert et al., 2008). The term “co-activation” has been introduced for experiments that use a Hebbian stimulation approach (Hebb, 1949). In this case, the simultaneous tactile “co-stimulation” of the skin is used to generate synchronous neural activity, which, according to Hebbian theory, is instrumental to drive plastic changes. The term “repetitive sensory stimulation” is often used for protocols that are independent of spatial cooperativity and use frequency and temporal patterning of stimulation. Other laboratories studying training-independent sensory learning use the framework of “tetanic” stimulation, which is commonly used in synaptic plasticity research (Clapp et al., 2005, 2012), or use the term “stimulus-selective response plasticity” (Cooke and Bear, 2012). More recently, “exposure-based learning” has been introduced to contrast feedback-induced learning with that generated by training via “exposure” to stimuli (Choi and Watanabe, 2012).

There is also confusion about the term “passive stimulation”: In the context of repetitive sensory stimulation experiments, this term indicates that subjects are exposed to sensory stimuli without attending actively to the stimulation, whereas in the framework of task-relevant training-based PL, “passive stimulation” is regarded as stimulation insufficient to drive learning processes. These examples indicate an obvious need for harmonization and standardization of terms used to characterize different forms of learning induction. Throughout this review we use the term “training-independent sensory learning” for learning induced by applying synaptic plasticity protocols in human participants with the aim of changing perception and behavior.

### A Hebbian learning protocol

In the “co-activation” stimulation protocol, the fingertip is repeatedly stimulated, either cutaneously or electrically, for many minutes to hours in order to induce plasticity in the corresponding primary and secondary somatosensory cortices (Godde et al., 2000; Pleger et al., 2001, 2003). Co-activation closely follows the idea of Hebbian learning: Synchronous neural activity is generated by simultaneous tactile “co-stimulation” of a large number of receptive fields. Because of the induced plasticity, tactile perception at the stimulated skin sites is altered. Spatial tactile discrimination, “tactile acuity”, is often assessed as a simple measure of changes in tactile perception abilities. In a typical co-activation experiment, two-point discrimination thresholds are lowered, indicating improved tactile acuity, which reaches baseline levels after 24 h (Godde et al., 2000). This co-activation-induced improvement does not transfer to fingers of the unstimulated hand, and there is no (or only weak) transfer to the neighboring fingers of the stimulated hand.

The relation between learning-induced changes in behavior and individual changes in brain organization has been studied using a combination of psychophysical tests and non-invasive imaging. Neuroimaging and electric source localization by multi-channel EEG showed that co-activation led to an increase in the size of the cortical representation specific to the co-activated finger (Pleger et al., 2001, 2003), which can be regarded as a recruitment of processing resources. The changes observed in cortical map representation were linearly related to the degree of improvement in two-point discrimination thresholds. Accordingly, a large gain in spatial discrimination abilities was associated with large changes in cortical maps (Pleger et al., 2001, 2003). Co-activation leads to re-organization of cortical functional networks that persists after seizing of stimulation in the so-called resting state (Freyer et al., 2012a).

Cellular studies have shown that increased excitability is a typical signature of effective LTP induction. In humans, so-called “paired-pulse stimulation”, the application of two stimuli in close succession, provides a reliable marker of excitability: The paired-pulse behavior is characterized by a significant suppression of the second response at short inter-stimulus intervals. Paired-pulse suppression was reduced after co-activation, and the amount of suppression was positively correlated with the individual gain in performance (Höffken et al., 2007). These data show that training-independent sensory learning results in selective reorganization of S1. These observations also suggest the important idea that the effect size differences typically observed across individuals reflect true differences in individual brain reorganization.

To demonstrate the Hebbian nature of the co-activation protocol, the effects of co-activation were compared to those of a so-called “single-site stimulation”, where only a small “point-like” skin area was stimulated. Stimulating the finger at a single site did not induce changes in discrimination performance or brain activity (Pleger et al., 2003) indicating a lack of brain reorganization and suggesting it is unlikely that other tasks beyond discrimination might have benefitted from single-site stimulation. It implies that a Hebbian “co-activation” is crucial for induction of plasticity effects and points to the requirement of spatial cooperative processes. Furthermore, the data emphasize that not all types of sensory stimulation lead to perceptual changes, and that there are “simple” forms of stimulation that remain ineffective in driving plasticity.

### LTP and LTD like sensory stimulation protocols

As outlined above, LTP and LTD are activity-dependent changes in the strength of synaptic connections, which are leading candidate mechanisms of neuronal plasticity. Therefore the efficacy of *in vitro* stimulation protocols in driving perceptual changes by applying high-frequency and low-frequency stimulation is of interest. High-frequency stimulation protocols consist of cutaneous pulse trains applied to the tip of the right index finger with a stimulation frequency of 20 Hz. Each train consists of 20 single pulses of 20 Hz lasting 1 s with an inter-train interval of 5 s. Low-frequency stimulation is applied at 1 Hz with stimulus trains consisting of 1200 pulses. Twenty minutes of high-frequency stimulation lower tactile discrimination threshold, whereas low-frequency stimulation results in an impaired discrimination performance. Most interestingly, 24 h after high-frequency stimulation, spatial two-point discrimination thresholds are still lower than the baseline values. In contrast, 24 h after low-frequency stimulation, the discrimination thresholds are recovered to the baseline values (Ragert et al., 2008). These results indicate that brief stimulation protocols resembling those used in cellular LTP and LTD studies can induce meaningful and persistent alterations in tactile discrimination behavior of humans.

### Neurotransmitters

Cellular studies have implicated the NMDA receptor as a major player in synaptic plasticity. A possible dependency of exposure-based learning on NMDA receptor activation was directly tested in humans using memantine, a substance blocking NMDA receptors selectively (Dinse et al., 2003). It was found that a single dose of memantine eliminated learning, both psychophysically and cortically, providing strong evidence of NMDA receptor involvement in training-independent sensory learning. Importantly, this finding implied that training-independent sensory learning is a plasticity-based process, which was debatable at that time.

While many drugs block learning, a few drugs are known to enhance cortical plasticity. *In vitro* experiments indicate that alterations in synaptic efficacy are induced by adrenergic agentsgating synaptic plasticity. In fact, a single dose of amphetamine (Dinse et al., 2003) results in almost a two-fold increase in both the normally observed improvement of tactile acuity and the cortical reorganization. These findings indicate that the processes underlying repetitive stimulation are further controlled by neuromodulatory systems (see Section Slow Oscillations to Coordinate System Consolidation of Hippocampal Memories in Sleep for the role of neuromodulation in hippocampus).

### Role of macroscopic brain states: resting state networks and alpha/beta band power fluctuations

From a global perspective learning arises from operations of adaptive networks. However, particularly the large-scale neural network level of learning is not well understood. On the macroscopic scale, top-down brain signals in distributed networks convey knowledge derived by prior experience. PET and fMRI have detected brain regions whose metabolic/vascular signal fluctuations correlate across time in task-free or “rest” settings (Raichle et al., 2001; Greicius et al., 2003; Fox et al., 2005). Those resting-state networks (RSN) are presumed to underlie sensory, motor, and cognitive functions. We will need to determine whether and how such large-scale neuronal dynamics regulate cognitive processes such as learning. Some evidence has already accumulated suggesting that intrinsic dynamics shape the manner in which we respond and adapt to external events which indicates that they might be relevant for learning. For example, it has been demonstrated that spontaneous activity fluctuations in the sensorimotor RSN account for a significant fraction of the trial-to-trial variability of the task-related fMRI response (Fox et al., 2006) and also contribute to trial-to-trial variability in button-press force (Fox et al., 2007).

Brain rhythms—such as EEG alpha (8–12 Hz) oscillations—are dynamical, complex signals with largely chaotic properties that can be described by nonlinear mathematical expressions (Freyer et al., 2009a, 2011a, 2012b). There is strong evidence that the alpha rhythm interacts with the processing of incoming stimuli for different sensory modalities (Nierhaus et al., 2009; Reinacher et al., 2009; Ritter and Becker, 2009). By creating mathematical models for the generation of visual evoked potentials, it has been demonstrated that the early-evoked response is compatible with the concept of linear superposition, whereas the late response (around 300 ms) is modulated by the strength of the preceding alpha rhythm (Becker et al., 2008). In line with this finding, the amplitude of late components of somatosensory evoked potentials depends on the amplitude of intrinsic alpha oscillations at time of stimulation (Reinacher et al., 2009). Variability of visually evoked fMRI responses is explained by ongoing alpha and its hemodynamic substrates (Becker et al., 2011). Ongoing alpha also determines the reaction times when subjects are asked to respond to specific stimuli (Becker et al., 2011). Another example is the predictability of human performance errors on the basis of preceding RSN activities (Eichele et al., 2008). Intriguingly even very subtle somatosensory evoked 600 Hz oscillations (ultra-high frequency oscillations, HFOs) with amplitudes in the order of few hundred nanovolt undergo spontaneous amplitude modulations paralleled by changes of the blood oxygen level dependent (BOLD) fMRI signal (Ritter et al., 2008b), see Section Role of Arrhythmic Fluctuations, High-Frequency Oscillations and the Gamma Band.

Studies from psychophysics indicate that intrinsic activity is responsible for human behavioral variance. Variability in human behavior often displays a 1/f frequency distribution with greater power at lower frequencies (Fox et al., 2007) corresponding to the spectral characteristics of endogenous BOLD and EEG signals. While fMRI can detect slow state modulations in the order of several seconds to minutes, EEG permits to explore both, fast oscillatory neuroelectric population activities with time constants below 1 s as well as slow activities. This makes simultaneous EEG-fMRI a promising approach for the exploration of the role of intrinsic activity for learning (Ritter and Villringer, 2006). It has been demonstrated that <1 Hz potential oscillations may mediate the hippocampal-neocortical dialog during the consolidation of explicit memories in sleep (Marshall et al., 2006). Learning not only crucially depends on oscillations but also in turn alters intrinsic brain rhythms (Huber et al., 2004; Mölle et al., 2004; Freyer et al., 2013). Brain rhythms and RSN dynamics are closely linked (Moosmann et al., 2003; Mantini et al., 2007; Ritter et al., 2008c; Reinacher et al., 2009). For example, numerous studies revealed a negative correlation between the strength of the posterior alpha rhythm and the fMRI signal in visual regions (Goldman et al., 2002; Moosmann et al., 2003; Gonçalves et al., 2006; de Munck et al., 2007), but see also (Laufs et al., 2003) and in sensorimotor regions (Ritter et al., 2009). A mechanistic biophysical understanding of this negative relation between rhythm strength and BOLD signal amplitude, however, has been lacking (Ritter et al., 2002). Yet a recent study constructing a biophysically plausible cortico-thalamic neural field model, reveals negative correlations between alpha oscillations and local population firing rates as likely source for the observed inverse relation between alpha and fMRI (Becker et al., under review). The cholinergic system is a possible neurotransmitter candidate for the link between mental states such as arousal and attention and rhythms and on the other hand is known to play an important role in learning (see Section also Slow Oscillations to Coordinate System Consolidation of Hippocampal Memories in Sleep, Interactions Between Intrinsic Activity and External Triggers in Perceptual Learning).

Though there exists the long-standing idea that learning can be facilitated by alpha or brain rhythm control via neurofeedback or entrainment, little sufficient evidence for these theories has been provided yet. Various neuronal populations in the brain can generate coherent states in a similar spectral range around 10 Hz. Hence alpha rhythms are not a unitary phenomenon but represent a large ensemble of integrative brain functions with different roles including for memory and learning (Klimesch, 1999). The primary site of plasticity in tactile PL (Fahle, 2002b; Seitz and Dinse, 2007) appears to be within the S1, an area exhibiting pronounced oscillatory activity (Salenius et al., 1997) and known for distinctive resting state dynamics (Beckmann et al., 2005). Hence the somatosensory system is a suitable system for exploring the role of intrinsic dynamics in learning. Recent studies combine empirical observations of EEG signals, psychophysics and computational models to identify biophysical mechanisms that link intrinsic brain activity with learning (Roy et al., 2014b).

The amplitude of spontaneous posterior alpha is negatively correlated with the fMRI signal in occipital areas and positively correlated in the thalamus (Moosmann et al., 2003). Correspondingly, local cortical deactivations at the generator sites of the more subtle 10 (alpha) and 20 Hz (beta) rolandic rhythms extracted by blind source separation have been reported. The alpha component was associated with postcentral deactivations (negative correlations with BOLD), whereas the beta component was associated with precentral deactivations (Ritter et al., 2009). The topographical differences support the theory of different functional roles of the two rhythms for somatosensory and motor processing respectively. These findings consistently indicate that cortical areas exhibiting spontaneous alpha and beta rhythms are deactivated—in the sense of an fMRI signal drop. Though the negative BOLD signal is still a matter of debate (Ritter et al., 2002), it has been shown that deactivations can occur during inhibitory processing (Wenzel et al., 2000; Blankenburg et al., 2003; Ritter and Villringer, 2006). Those results support the theory that background rhythms in the alpha and beta frequency range are indicators of a balance shift towards inhibitory processing. Recent findings though suggest that alpha and beta background rhythms may further play an active role by facilitating Hebbian learning and phase coding (Roy et al., 2014b; Sigala et al., 2014).

#### Role of arrhythmic fluctuations, high- frequency oscillations and the gamma band

Brain states are also indexed by arrhythmic fluctuations. Direct current (DC) potential studies show that the phase of slow cortical potentials modulates higher frequency activities (Rebert, 1973; Vanhatalo et al., 2004; He et al., 2010) and performance, e.g., (Born et al., 1982; Stamm et al., 1987). Several recent studies show that transcranial direct current stimulation (tDCS) may potentially enhance learning. A postulated mechanism is the known modulation of DC potentials by tDCS (Elbert et al., 1981a,b; Marshall et al., 2004). The arrhythmic fluctuations of broadband high-gamma power could also be an important indicator of brain states, given its tight correlation to neuronal firing rate (Ray and Maunsell, 2011; Manning et al., 2012).

Communication between neurons is reflected by action potentials. In EEG traces of population spikes can be recorded under certain conditions (Baker et al., 2003). It is a great challenge though to measure these subtle 600 Hz signatures during simulatneous EEG-fMRI since high-amplitude artifacts distort the EEGs during fMRI acquisition. Yet with optimized setups and artifact correction procedures (Becker et al., 2005; Ritter et al., 2007, 2010; Freyer et al., 2009b) reliable measures of 600-Hz population spikes can be obtained. Spontaneous dynamics of these HFOs revealed distinct fMRI activation sites along the thalamocortical pathway for early and late bursts separated in time by only few milliseconds (Ritter et al., 2008a). The theory that endogenous rhythms are relevant for information processing and memory storage is increasingly accepted, however consistent results with respect to the underlying mechanisms are still missing.

Studying brain activity during the development of the learning processes brings new insights about the role of brain states for learning. In a recent study Hamamé et al. (2012) looked at the final effect of training in a visual perceptual task, but they also correlated brain activity with different PL epochs. Hamamé et al. observed that oscillatory activity in both, the alpha and gamma bands, was present during the learning acquisition process. Previously, gamma but not alpha band activity has been found to correlate with learning in studies that compared pre- vs. post training or trained vs. non-trained subjects (Miltner et al., 1999; Gruber and Müller, 2002; Gruber et al., 2002).

#### Variability of perceptual learning outcome

An interesting phenomenon in PL is the great variability of the learning outcome across subjects (Fahle et al., 1995; Fahle and Henke-Fahle, 1996; Hodzic et al., 2004). A recent study has shown that resting state alpha activity interacts with incoming stimuli to shape PL in the somatosensory modality (Freyer et al., 2013). Freyer et al. (2013) investigated to which extend the different ongoing neuronal states of the individual subjects, before and during repetitive sensory stimulation/coactivation, explain the observed differences in the learning success. They found that up to 64% of the observed variability is predicted by: (1) pre-learning parietal alpha, representing an idling state potentially linked to the default mode network; and (2) stimulus-induced contralateral central alpha changes, indicative for the degree of engagement of sensorimotor areas during training (Freyer et al., 2013). These findings also indicate that the readiness and efficacy to process incoming sensory stimuli plays a major role in permitting PL to occur. For detailed discussion about possible mechanisms underlying the observed alpha rhythm state-dependencies in PL see Sigala et al. (2014).

## Translation to clinics

### Neuroplasticity based rehabilitation in brain injury and stroke

Paradigms used to decipher the state-dependencies of learning enable a translation of recent research to clinics. Neuroplasticity-based rehabilitation after brain injury and stroke typically uses task-specific training and massed practice to drive brain reorganization and improve sensorimotor functions (Taub et al., 2002). As many patients have restricted mobility, however, the development of additional approaches that may supplement, enhance or even replace conventional training procedures would be of advantage. Therefore, the feasibility of repetitive sensory stimulation approaches is been increasingly explored (Dinse et al., 2005, 2011; Hummel and Cohen, 2006; Wu et al., 2006; Kattenstroth et al., 2012). Available data suggest that application of training-independent sensory learning to patients with brain injury provides a surprisingly effective way to ameliorate perceptual and behavioral impairments (de Kroon et al., 2002; Hummel and Cohen, 2006; Sawaki et al., 2006; Wu et al., 2006; Smith et al., 2009; Kattenstroth et al., 2012). The particular advantage of training-independent sensory learning is its passive nature, which does not require the active participation or attention of subjects (see Section Exposure Based Learning). Therefore, training-independent sensory learning approaches can be applied in parallel with other techniques, which makes this intervention very easy to implement and more acceptable to the individual.

### Neuroplasticity to counteract age related impairments

The same rationale holds true for the treatment of age-related impairments, although an appreciation of the urgent need of treatment of age-related degradation compared to that of impairments after brain injury is less apparent. Nonetheless, given the dramatic changes in the age structure of industrialized societies, substantial efforts are currently being undertaken to improve cognition and sensorimotor performance of the elderly by training, exercising, and practicing (Kramer and Erickson, 2007). Some recent studies on elderly individuals showed improvement in tactile and sensorimotor performances (Dinse, 2006; Dinse et al., 2006) and stabilized recovery, over repeated applications of training-independent sensory learning (Kalisch et al., 2010). These data suggest that training-independent sensory learning is also effective in aged populations characterized by severe impairments of perception and sensorimotor behavior.

### Neurofeedback learning

A challenging active learning technique is neurofeedback, which requires acquisition analysis and feedback of brain signals (e.g., fMRI, M/EEG, optical signals) in real-time. Brain responses are continuously monitored, translated into a perceivable—e.g., visual, acoustic or tactile—signal, and back-projected to participants who, over the course of typically several training sessions, learn to consciously regulate their brain activity. An extensive body of literature starting in the 1970s focused on neurofeedback training. Such training was implemented to control various EEG measures, providing evidence of positive effects in patients with otherwise pharmacologically intractable epilepsy, attention deficit disorder, and hyperactivity (Birbaumer et al., 2009).

Real-time fMRI is a more recent technique for assessing the dynamic and robust changes in the brain’s hemodynamic activity during ongoing experiments. The extracted information serves as basis for neurofeedback training. Since its implementation methodological and conceptual limitations were substantially reduced by artifact control, sensitivity improvements, real-time algorithms, and adapted experimental designs (Weiskopf et al., 2004; Bagarinao et al., 2006). Physiological self-regulation of the local hemodynamic response via neurofeedback is a challenging paradigm for cognitive neuroscience to induce behavioral alterations in combination with brain plasticity. Results so far have demonstrated a variety of potential applications for real-time fMRI also for clinical use, such as strengthening cognitive control over pain (Weiskopf et al., 2004). Real-time fMRI based neurofeedback training enables healthy subjects to consciously modulate neural activity in posterior insular cortex, a brain region known to be involved in interoceptive awareness, i.e., the sense of the physiological condition of the body (Craig, 2003). Dijkerman and de Haan formulated a somatosensory model of perception and action (Dijkerman and de Haan, 2007), mimicking other sensory domains such as the visual or auditory one. Their model conceptualizes two cortical pathways. The action-associated dorsal pathway is assumed to project from postcentral gyrus via parietal operculum to the posterior parietal cortex. The ventral pathway instead subserves recognition and perception and terminates in the anterior and posterior insular cortex after passing postcentral gyrus and parietal operculum. In agreement with this assumption of a central role of posterior insula in tactile perception and recognition, the same structure has been identified (Pleger et al., 2006) as being involved in a widely used somatosensory frequency discrimination task (Romo and Salinas, 2003). In this working-memory task, on each trial, two stimulations of different frequencies, separated by a few seconds, are presented to a fingertip. Subjects have to decide whether the first or the second stimulation contains the higher frequency. This process comprises three different processing steps: encoding, maintaining, and decision-making, all requiring interoceptive capacities. Stimulus encoding involves primary sensory brain regions. For maintaining the first stimulation until the second is presented, the first one must be temporarily stored within prefrontal cortex. The decision process, starting with presentation of the second stimulation, requires information exchange between secondary somatosensory cortex representing the second stimulation and prefrontal cortex memorizing the first one (Romo et al., 2002; Machens et al., 2005). Subjects’ individual frequency discrimination abilities can be tested in a classic event-related fMRI experiment (Pleger et al., 2006) after each neurofeedback training session to unveil training influences on the somatosensory cortex and interconnected cognitive regions in prefrontal cortex. Gaining control over activity in posterior insula may either improve the encoding in primary and secondary somatosensory cortex and/or the maintenance/decision-process involving prefrontal and secondary somatosensory cortex. An improvement in frequency discrimination after neurofeedback training may therefore either be accompanied by enhanced activation and connectivity within somatosensory cortices and/or in prefrontal and secondary somatosensory cortex. These combined behavioral and functional cortical changes may parallel distinct structural alterations of posterior insular cortex, possibly together with interconnected somatosensory/prefrontal regions. These are of specific interest since they represent consolidation processes of rather unspecific neurofeedback training on interoceptive awareness with its specific influences on well-defined somatosensory-cognitive functions (encoding to decision-making).

An increasing number of clinical applications of neurofeedback training for a variety of pathologies are currently under investigation. Among others, effects have recently been discussed for the treatment of attention-deficit/hyperactivity disorder (Duric et al., 2012), migraine (Stokes and Lappin, 2010), tinnitus (Busse et al., 2008), and the assistance of post-stroke motor rehabilitation (Cincotti et al., 2012).

Our future challenges are to combine the different methods and findings delineated above for a better understanding of state-dependencies of learning across brain scales. For instance, comparing coactivation-induced changes in hemodynamic and EEG background rhythm with recordings obtained over the course of the coactivation procedure (see Section Role of Macroscopic Brain States: Resting State Networks and Alpha/Beta Band Power Fluctuations) will be of particular interest for the understanding of how coactivation-induced behavioral and cortical changes mature over the course of the application and to identify crucial neurobiological states relevant for an optimal coactivation influence (e.g., certain EEG background rhythms). This allows to specifically trigger these states, e.g., by means of neurofeedback training or other interventions such as non-invasive brain stimulation, in order to boost coactivation-induced plasticity. The overarching goal is to identify brain states specifically sensitive to plasticity-driving interventions. The identification of such spatial or temporal states may in future help to develop new treatment options for brain injury. One possible research strategy is to investigate functional/structural brain plasticity and behavioral recovery after brain injury over time. Understanding the temporal dynamics of recovery would allow to specifically assigning appropriate neurorehabilitative interventions to certain temporal states of recovery to further optimize patients’ outcome.

Another novel approach to neurofeedback learning was undertaken recently (Kovacevic et al., under review). During an art festival data from over 500 people were collected. In a collective computer game participants controlled their mental states of relaxation and concentration with EEG neurofeedback. Learning related changes in the EEG power spectrum were detectable already after a short training period of about one minute. In addition baseline brain activity predicted the training outcome indicating state-dependent learning. Such quick training effects are relevant for every-day brain-computer interfaces (BCI) applications. The study indicates a novel avenue of application-oriented neuroscience fostering the understanding of brain functionality under natural conditions—rather then laboratory settings—by linking art and science.

## Conclusions and outlook

The molecular principles of synaptic plasticity are relatively well understood. Yet the principles that orchestrate plasticity to render it a meaningful cognitive instrument are less clear. The brain continuously reorganizes thereby consolidating new memories while maintaining or erasing old ones. Understanding the underlying complex principles of interactions between the intrinsic functional connectivity (i.e., intrinsic brain states) and external triggers stimulating the brain is essential for our understanding of learning. Revealing those principles is important for clinical applications and also for the development of novel neuromorphic technologies, for a comprehensive account see two special issues of the BrainModes conference series (Breakspear et al., 2009; Terry et al., 2011). Our review is capturing just some aspects of learning. Integration of a vast amount of knowledge is necessary to solve the riddle. Important achieved milestones are the possibility to derive activity from inside a neuron in a behaving animal and the possibility to integrate large-scale observations—such as with EEG and fMRI—in a biophysically plausible full brain models such as The Virtual Brain. Adding detail to large-scale full brain models, such as implementing plasticity rules and receptor distributions, will help to incrementally increase our understanding of potential mechanisms underlying learning. This implies an iterative process between theoretical and empirical neuroscience at macro, meso and microscopic brain scales and hence calls for collaborations between fields and for standardization/unification in several respects including terminology, mathematical framework and brain data—both simulated and real.

## Conflict of interest statement

The authors declare that the research was conducted in the absence of any commercial or financial relationships that could be construed as a potential conflict of interest.

## Abbreviations

ACh: Acetylcholine
BOLD: Blood oxygen level dependent
CA: Cornu Ammonis of hippocampus
DC: Direct current
EC: Entorhinal cortex
EEG: Electroencephalography
fMRI: functional magnetic resonance imaging
HFO: High-frequency oscillation
LTD: Long-term depression
LTP: Long-term potentiation
MEG: Magnetencephalography
NE: Norepinephrine
NMDA: N-methyl-D-aspartate
PL: Perceptual learning
REM: Rapid eye movement
RSN: Resting state network
S1: Primary somatosensory cortex
SO: Slow oscillations
SPW-Rs: Sharp wave ripples
STDP: Spike-timing dependent plasticity
SWS: Slow-wave sleep
tDCS: Transcranial direct current stimulation.
